# Bariatric Endocrinology: Principles of Medical Practice

**DOI:** 10.1155/2014/917813

**Published:** 2014-05-12

**Authors:** J. Michael Gonzalez-Campoy, Bruce Richardson, Conor Richardson, David Gonzalez-Cameron, Ayesha Ebrahim, Pamela Strobel, Tiphani Martinez, Beth Blaha, Maria Ransom, Jessica Quinonez-Weislow, Andrea Pierson, Miguel Gonzalez Ahumada

**Affiliations:** Minnesota Center for Obesity, Metabolism and Endocrinology (MNCOME), 1185 Town Centre Drive, Suite 220, Eagan, MN 55123, USA

## Abstract

Obesity, is a chronic, biological, preventable, and treatable disease. The accumulation of fat mass causes physical changes (adiposity), metabolic and hormonal changes due to adipose tissue dysfunction (adiposopathy), and psychological changes. Bariatric endocrinology was conceived from the need to address the neuro-endocrinological derangements that are associated with adiposopathy, and from the need to broaden the scope of the management of its complications. In addition to the well-established metabolic complications of overweight and obesity, adiposopathy leads to hyperinsulinemia, hyperleptinemia, hypoadiponectinemia, dysregulation of gut peptides including GLP-1 and ghrelin, the development of an inflammatory milieu, and the strong risk of vascular disease. Therapy for adiposopathy hinges on effectively lowering the ratio of orexigenic to anorexigenic signals reaching the the hypothalamus and other relevant brain regions, favoring a lower caloric intake. Adiposopathy, overweight and obesity should be treated indefinitely with the specific aims to reduce fat mass for the adiposity complications, and to normalize adipose tissue function for the adiposopathic complications. This paper defines the principles of medical practice in bariatric endocrinology—the treatment of overweight and obesity as means to treat adiposopathy and its accompanying metabolic and hormonal derangements.

## 1. Introduction

Overweight and obesity are a continuum, and together they represent a chronic, biological, preventable, and treatable disease. Overweight and obesity are an epidemic affecting two thirds of the American population [1–3]. The accumulation of fat mass leads to physical changes (adiposity), metabolic changes due to adipose tissue dysfunction (adiposopathy), and psychological changes, each of which adversely affects health (Table 1) [4].

Obesity has long been held a risk factor for diabetes, hypertension, dyslipidemia, and premature cardiovascular disease [5–8]. The strong association between intra-abdominal, visceral fat and metabolic disorders led the National Cholesterol Education Program Expert Panel on Detection, Evaluation and Treatment of High Blood Cholesterol in Adults, Adult Treatment Panel III (NCEP-ATP III) to include the measurement of waist circumference as a defining element of the dysmetabolic syndrome [9, 10]. In the NCEP-ATP III initial report, the five elements that defined dysmetabolic syndrome included a waist circumference (≥40 inches in men and ≥35 inches in women), high blood pressure (≥130/85), high fasting plasma glucose concentration (≥110 mg/dL), hypertriglyceridemia (≥150 mg/dL), and low plasma levels of high density lipoprotein cholesterol (HDL-C) (<40 mg/dL in men and <50 mg/dL in women). Three or more of these five diagnostic criteria give an individual the diagnosis of dysmetabolic syndrome, for which the International Classification of Diseases, 9th edition, introduced the code 277.7. Following this report, intra-abdominal or visceral fat became a treatment target in medical practice.

Adipose tissue dysfunction is etiological in the development of the metabolic and endocrine derangements that accompany overweight and obesity. This concept is now well established in the medical literature [8, 11–13]. What follows from acceptance of this premise is near-universal agreement that early weight loss is a desirable part of medical treatment, best implemented prior to the development of complications with irreversible health consequences. The observation that in some cases overweight and obesity are associated with better health outcomes, especially as it relates to vascular disease, the so-called “obesity paradox,” has subtracted from the sentiment that overweight and obesity should be treated [14–19]. The obesity paradox is likely due to having better lean mass index and cardiovascular fitness in some people with overweight and obesity, and to differences in treatment. Patients with overweight and obesity that have had vascular events are treated more aggressively than thin patients [17–20]. The “obesity paradox” aside, the formal implementation of a long-standing weight management program that includes pharmacological intervention is still missing from medical practice. Even contemporary reviews by leaders in the field of obesity lack this important element of medical practice. The discussion of the management of overweight and obesity frequently jumps from lifestyle changes to bariatric surgery, and the lack of data on the long-term use of pharmacotherapy is the only mention given to this subject [21, 22].

The drivers for food intake are complex, and hedonic food control plays a major role for many people [23–25]. The hypothalamus is the major control center for energy balance and adipose tissue stores, but other brain centers also play a role [26–29]. Therefore, therapy for adiposopathy hinges on effectively lowering the ratio of orexigenic to anorexigenic signals reaching the the hypothalamus and other relevant brain regions, favoring a lower caloric intake.

Effective reductions of body fat (including intra-abdominal fat) are accomplished by an integrated, team approach to patient care [11]. Adiposopathy, overweight and obesity should be treated indefinitely with the specific aims to reduce fat mass for the adiposity complications, and to normalize adipose tissue function for the adiposopathic complications.

This paper defines the principles of medical practice in bariatric endocrinology—the treatment of overweight and obesity as means to treat adiposopathy and its accompanying metabolic and hormonal derangements.

## 2. Principle 1—Overweight and Obesity Are a Continuum and Together Represent a Chronic, Biological, Preventable, and Treatable Disease

Adipose tissue is distributed throughout the human body. Adipose tissue is metabolically active, both receiving and sending signals that help regulate metabolism [30–33]. Adiposity, the physical accumulation of fat mass through fat hypertrophy or fat hyperplasia, leads to many mechanical complications that need to be identified and treated, since they are obstacles to effective weight loss. Among these are degenerative osteoarthritis and obstructive sleep apnea [34–39]. The accumulation of fat mass is also strongly associated with psychiatric disease [40–45]. Additionally, psychotropic medications are associated with metabolic diseases (Table 2) [46–49]. Simply on the basis of physical and psychological complications, overweight and obesity meet the definition of a chronic disease.

Overweight and obesity are commonly defined by the body mass index (BMI)—calculated by dividing body weight in kilograms by height in meters squared [50]. Commonly used BMI cutoff values to diagnose obesity have high specificity but low sensitivity to identify adiposity, as they fail to identify half of the people with excess body fat percentage [51]. Thus, newer approaches that seek to quantitate different fat pools and differentiate between lean and fat mass, including DXA body composition analysis, may have more clinical relevance [8, 52–57].

In clinical practice, body weight, waist circumference, and waist-to-hip ratio provide sufficient assessment of metabolic risk. These measurements are best interpreted as they evolve over time. When possible, the differentiation between lean and fat mass is desirable, especially to find individuals who have significant visceral fat mass, but whose BMI might not place them in an overweight or obesity category.

**Figure 1:**
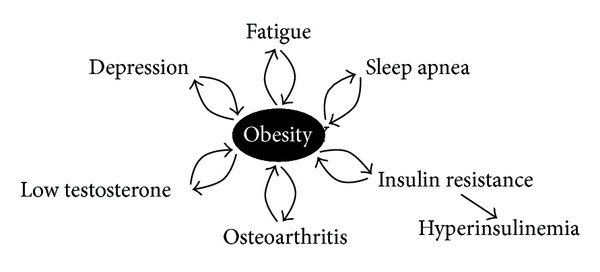
Complications of obesity that interfere with its treatment (Copyright © Minnesota Center for Obesity, Metabolism and Endocrinology, PA).

Adiposopathy is a complication of excess fat mass when adipose tissue becomes dysfunctional. Bariatric endocrinology takes into account the physical and psychological complications of overweight and obesity and the role they may play as obstacles to effective weight loss and metabolic health (Figure 1).

## 3. Principle 2—Every Patient Who Has Overweight or Obesity Should Be Initially Evaluated for Causes and Complications of Weight Gain, Including Adiposopathy

Adipose tissue is not just for the storage of energy. Rather, it is a very active endocrine and metabolic tissue, and it actively helps regulate metabolism. Like any other tissue, adipose tissue may change anatomically, and it may become dysfunctional. The concept that altered anatomy and function of adipose tissue cause metabolic diseases is now well engrained in the medical literature [58–61]. Cardiomyopathy, myopathy, encephalopathy, ophthalmopathy, retinopathy, enteropathy, nephropathy, neuropathy, and dermopathy define disease of specific tissues or organs. Similarly, adiposopathy defines disease of adipose tissue. To continue the analogy, in cardiomyopathy there is enlargement of cardiac myocytes (hypertrophy), which leads to clinical disease (cardiac outlet obstruction). And in adiposopathy there is enlargement of fat cells, leading to clinical disease. Adiposopathy is also anatomically manifested by visceral adiposity, growth of adipose tissue beyond its vascular supply leading to ischemia and inflammation, increased number of adipose tissue immune cells, and ectopic fat deposition (in other body tissues, i.e., fatty liver) [8, 13].

Adiposopathy is functionally manifested by: [12, 13]

- impaired adipogenesis with the subsequent development of adipocyte hypertrophy [64–66],
- heterogeneous distribution of adipose tissue (e.g., visceral adiposity),
- adipocyte lipolysis in excess of lipogenesis leading to increased free fatty acid release into the circulation,
- pathogenic adipose tissue endocrine responses (e.g., hypoadiponectinemia and hyperleptinemia),
- pathogenic adipose tissue immune responses,
- pathogenic “crosstalk” between adipose tissue and other organs [13, 67].

The adipose tissue changes of adiposopathy affect other tissues, changing homeostasis and leading to derangements of metabolism (e.g., insulin resistance with eventual hyperglycemia, hypogonadism, hepatosteatosis possibly leading to steatohepatitis, etc.) [61] and increased cardiovascular risk [8, 68, 69].

For all of the above reasons, in the evaluation and management of patients with overweight and obesity there must be a thorough evaluation looking for causes and complications of the disease, including a complete history and physical examination and laboratory testing [21, 70–72].  Table 3 lists medications that are commonly associated with weight gain, which should be substituted for as possible.  Table 4 lists the laboratory findings that may be found in patients with adiposopathy.

## 4. Principle 3—Every Patient Who Has Overweight or Obesity Should Have Periodic Risk Restratification

Not all patients with an elevated BMI have adiposopathy and metabolic diseases. And not all patients who have adiposopathy and metabolic diseases have an elevated BMI (Table 5) [62]. For this reason, it is imperative that patients have a periodic re-stratification of their metabolic risk. In our practice we recommend that all patients who have overweight or obesity by their BMI have a yearly risk re-stratification with laboratory testing (Table 4).

## 5. Principle 4—There Are No Short-Term Solutions to a Chronic Medical Problem: Overweight and Obesity Should Be Treated with the Same Model of Chronic Disease Management That We Use for Other Chronic Diseases

No one would think of treating diabetes for just a few weeks or months. No one would think of stopping treatment if initial monotherapy for diabetes did not result in the expected reductions in hemoglobin A_1c_. And no one would ever think of treating congestive heart failure, hypertension, epilepsy, asthma, multiple sclerosis, hypothyroidism, or any other chronic disease, for just a short while. Yet, overweight and obesity are not held in the same light. Historically, treatments for obesity were limited in duration. The genesis of this restriction in duration of treatment was the fear that centrally acting medications might have addictive potential or lead to tolerance. Because of these government agency restrictions, the prevailing attitude in the medical community has been that overweight and obesity are not treatable as a chronic disease.

The last five years have brought about a change in this attitude. Several leading organizations including the American Association of Clinical Endocrinologists [73], the National Lipid Association [8], the Obesity Society/American College of Cardiology/American Heart Association [21], the American Society of Bariatric Physicians [74], and the National Diabetes Education Program [75] have all issued statements calling for the treatment of obesity as a chronic disease. And the American Medical Association (AMA) finally recognized obesity as a chronic disease at the annual meeting of the House of Delegates in Chicago, in June 2013.

The approach to overweight and obesity should be exactly the same as the approach to any other chronic disease. Using diabetes as an example, the management of overweight and obesity over time should follow a parallel track (Table 6).

## 6. Principle 5—Effective Behavior Modification to Achieve a Negative Energy Balance Is the Primary Long-Term Goal of the Medical Treatment of Overweight and Obesity

The hypothalamus and other relevant brain regions regulate the frequency and volume of feedings [26–28, 76–79]. This is a highly complex process that takes input from throughout the body, and carefully determines the balance of orexigenic and anorexigenic stimuli both in health and disease [80–87]. The primary long-term goal of the medical management of overweight and obesity is to reset the hypothalamus and other relevant brain regions to allow for satiety at a lower set point.

Behavior modification to achieve a lower caloric intake and an increased level of physical activity (caloric expenditure) should be implemented and maintained. Patients must learn how to achieve these goals. It is not enough to tell a patient that they should restrict calories and become more active. They must be taught* how* to achieve these goals as explicitly as possible.

Simple concepts are important to deliver in patient education and need to be reviewed repeatedly.

- It is possible to achieve fullness with fewer calories by increasing the volume of meals with foods that have a lower caloric density [11, 88, 89].
- Drink water instead of high-calories beverages—a twist of lime makes it much more palatable.
- Using a smaller diameter plate for meals makes portions smaller (i.e., the MyPlate campaign by the FDA) [11, 90–92].
- It should be a goal to ingest 10 servings of fresh fruits or vegetables every day [11].
- Caloric expenditure is accomplished by moving body mass over distance or against gravity. It does not have to be done fast, or all at once. A two-minute walk every hour on the hour during the waking hours of the day is equivalent to a 30-minute walk [8].
- Avoid the words “diet” and “exercise” and focus on healthy eating and meal planning and on physical activity that is achievable, realistic, sustainable, and incrementable.
- Quantitate physical activity with tools such as a pedometer.
- Involve your support structures at home and at work.
- Push yourself to be physically active when you get hungry. Going up and down two flights of stairs instead of going to the kitchen for food will make it unlikely that hunger prevails.

All of these examples of behavior modification have the common goal of decreasing orexigenic stimuli, and increasing anorexigenic signals to the hypothalamus and other relevant brain regions. It takes time, but by achieving consistent lifestyle changes, the signals that reach the hypothalamus and other relevant brain regions will change to favor a lower set point for energy balance.

## 7. Principle 6—The Team Approach to Overweight and Obesity Should Be Offered to All Patients to Provide Nutrition Education and Physical Activity Coaching

Obesity lends itself to the model of chronic endocrine disease management that is commonly accepted for other chronic diseases, such as diabetes (Table 6) [63]. Implicit in this model is a comprehensive team approach to patient care with an emphasis on patient education [93–103]. Also implicit in this model of care is the existence of the infrastructure to make patients comfortable—wide chairs, large gowns, scales that accurately measure up to 800 pounds, large blood pressure cuffs, contingency plans that take into account very large body mass (e.g., preparedness to handle a syncopal episode or a cardiac arrest of a very large person), and staff that has been trained to be understanding and supportive.

Central to patient education and support is the availability of materials to meet individual patient needs. Props and models, electronic media, print media, audiovisual resources, web-based education, and group classes are all viable means to provide education.

The health care team should include specialized clinicians, nurse educators, dietitians, behavioral counselors, mental health professionals, pulmonologists, physical medicine and rehabilitation consultants, physical therapists, pain specialists, dermatologists, gynecologists, oncologists, cardiologists, bariatric surgeons, and others.

## 8. Principle 7—Pharmacotherapy Should Be Used Indefinitely in the Management of Overweight and Obesity

“It is possible to frame a house with a bucket of nails and a hammer. But it is faster and better if one gets an air compressor and an air gun.” This message resonates with patients when introducing the concept of pharmacotherapy for obesity. Pharmacotherapy significantly enhances the beneficial changes of better nutrition and increased physical activity.

Medications for obesity have a tainted past. When amphetamines were used as weight loss agents and their use became popular in the 1950s, it soon became evident that they are habit-forming, tolerance-building, and addictive. Subsequent centrally acting medications were all labelled as having the potential for addictiveness. Because of this concern, and because obesity was considered a condition and not a disease back then, medications for weight loss were approved only for short-term use.

Most recently, rimonabant, which was effective in achieving weight loss, was withdrawn from the market in 2009 because of an association with depression and suicides [104, 105]. Additionally, sibutramine was removed from the market in 2010 because it was not superior to placebo in decreasing vascular events in a population at risk [106–108], and because in the Sibutramine Cardiovascular Outcomes Trial (SCOUT) it was shown to have increased mortality in patients with established arteriosclerosis. These recent developments followed the association of fenfluramine use with valvular heart lesions in the 1990s, leading to an abrupt end to the use of fenfluramine with phentermine (fen-phen) and removal of fenfluramine and dexfenfluramine from the market.

The popular notion that pharmacotherapy for obesity carries a higher risk than benefit, and that the historical restrictions on length of treatment, are proof in practice of this risk, is pervasive in medicine. Indeed, it is pervasive in society. For example, Minnesota has a statute that makes it illegal for the state to pay for any obesity medications.

Thus, pharmacotherapy for obesity will need strong data to support its long-term use. In the meantime, a lack of such data is* not* a reason not to treat patients who have a serious, chronic disease.

The initial approach to overweight and obesity should always be to focus on lifestyle changes. The vast majority of patients will benefit from pharmacotherapy. Initially monotherapy with any one of a number of available agents is appropriate. Economics frequently dictates which agent will be used. Of all obesity medications, generic phentermine is the least expensive and is available, cash-out-of-pocket, for as little as $10.00 USD a month. Other medications for monotherapy will have incremental cost.

Of note, all medications currently approved for overweight and obesity in the United States are pregnancy category X, where the FDA considers the risks involved in use of the drug in pregnant women to clearly outweigh potential benefits. Paradoxically, pregnancy in some women may only be achieved with insulin sensitization and weight loss.

Phentermine, available since the 1950s, is indicated for short-term use, but it continues to be used as an “off-label” single agent for prolonged treatment of overweight and obesity. Phentermine is a trace amine-associated receptor 1 (TAAR1) agonist. TAAR1 is an amine-activated Gs and Gq protein-coupled receptor located at the neural presynaptic membrane [109]. Although the precise mechanism of action of phentermine on the hypothalamus and other relevant brain regions remains unknown, it causes an anorexigenic effect [110–113]. Phentermine also increases adrenergic tone, leading to adrenal release of catecholamines, which causes lipolysis. This adrenergic effect of phentermine mandates careful monitoring of the blood pressure. At clinically relevant doses, phentermine also releases serotonin and dopamine, but to a much lesser extent than norepinephrine [114]. Phentermine is not habit-forming, tolerance-building, or addictive. Discontinuation of phentermine leads to a loss of therapeutic effect and no other untoward effects [115]. Phentermine in combination with topiramate may be used as initial therapy for overweight and obesity and is discussed below.

It is important to note that neither phentermine nor any other currently available centrally acting agents take away a patient's appetite. Appetite is a survival mechanism that ensures a continuous energy supply for the organism's needs and is preserved with the use of phentermine and other weight loss drugs.

Orlistat is a pancreatic lipase inhibitor. By preventing fat break-down during digestion, orlistat causes fat malabsorption and weight loss. The potential side effects of this medication are mostly due to steatorrhea. Anecdotal reports of liver damage and acute oxalate nephropathy have been reported with orlistat use. Orlistat leads to significantly more weight loss than placebo, with the ensuing beneficial metabolic changes associated with it [116–118]. With orlistat treatment 35.5 to 54.8% of patients achieve ≥5% weight loss and 16.4 to 24.8% of patients achieve ≥10% weight loss after one year of treatment [119, 120]. Following 4 years of treatment with orlistat, 28% of the placebo patients and 45% of the orlistat patients lost ≥5% of their baseline body weight and 10% of the placebo patients and 21% of the orlistat patients lost ≥10% of their baseline body weight [116, 117].

Lorcaserin is a selective 5-hydroxytryptamine T_2C_ receptor agonist which promotes weight loss through satiety [121, 122]. The dose of lorcaserin is one 10 mg tablet twice daily. It was approved for the treatment of obesity in the United States in 2012. There are three registration trials for lorcaserin: Behavioral Modification and Lorcaserin for Overweight and Obesity Management (BLOOM) trial [123], Behavioral Modification and Lorcaserin Second Study for Obesity Management (BLOSSOM) trial [124], and the Behavioral Modification and Lorcaserin for Overweight and Obesity Management in Diabetes Mellitus (BLOOM-DM) trial [125]. In comparison to placebo, lorcaserin decreased waist circumference, blood pressure, total cholesterol, low-density lipoprotein-cholesterol and triglycerides. The most frequent adverse events reported with lorcaserin are headache, dizziness, and nausea [126]. Lorcaserin did not statistically affect heart rate, like phentermine does, or high-density lipoprotein-cholesterol [126].

Three other centrally acting drugs are currently FDA-approved for treatment of obesity: benzphetamine, phendimetrazine, and diethylpropion. All three are centrally acting medications that cause early satiety and have similar potential side effects to phentermine.

Bupropion, fluoxetine, and other antidepressants are sometimes useful in the management of obesity. Antidepressants have significant variability on weight. It remains unclear if the effect of antidepressants on weight is due to the medication itself or the treatment of the underlying depression. Some patients lose weight with resolution of depression, and some gain weight.

Other medications that are associated with weight loss include metformin, exenatide, liraglutide, pramlintide, canagliflozin, dapagliflozin, and topiramate monotherapy. None has an approved indication for weight loss.

## 9. Principle 8—Failure of Monotherapy to Achieve Effective Weight Loss Should Not Lead to Discontinuation of Treatment, but Rather to the Institution of Combination Therapy

Combination therapy for obesity has been an option for decades. Fen-phen, discussed above, helped thousands of patients lose weight and was a prime example of the benefit of combination therapy.

The US Food and Drug Administration requires that the labeling for obesity medications, including orlistat and lorcaserin, calls for discontinuation of these medication if 5% weight loss is not achieved after 12 weeks of treatment. The authors strongly disagree with this approach to the management of overweight, obesity, and adiposopathy. As is the case with diabetes mellitus, dyslipidemia, hypertension, heart failure, asthma, depression, and every other chronic disease, the initial agent used for weight loss monotherapy is unlikely to achieve a full therapeutic goal, even with dose titrations. For this reason, the authors strongly recommend that for overweight, obesity, and adiposopathy, as is the case with other chronic diseases, monotherapy should be continued, and combination therapy should be instituted when the treatment effect of one agent wanes or is insufficient to reach treatment goals. The 5% threshold set by the FDA is arbitrary, especially when one considers that lack of treatment might have led to continued weight gain. Compared to no treatment even those who do not reach the arbitrary 5% threshold benefit from pharmacotherapy. Of note, until the data is generated, prolonged use of older medications, and combinations of medications, other than phentermine-topiramate, which is already in the market, will have to be “off label.”

Currently, the only combination product that is available in the United States market is Qsymia, a combination of phentermine with topiramate approved for treatment of obesity in 2012. There are three registration trials: EQUIP [127], EQUATE [128], and CONQUER [129]. Qsymia causes significant weight loss, reduction in waist circumference and improvements in glycemia, lipids, inflammatory markers, and blood pressure. The recommended initial dose is 3.75 mg/23 mg (phentermine 3.75 mg/topiramate 23 mg extended release) daily for 14 days and then increased to 7.5 mg/46 mg daily. If 3% weight loss is not achieved after 12 weeks on the 7.5 mg/46 mg dose, escalation to the maximum dose of 15 mg/92 mg is recommended. The 7.5 mg/46 mg dose should not be exceeded for patients with moderate or severe renal impairment or patients with moderate hepatic impairment. Adverse events of Qsymia included paresthesias, dry mouth, constipation, dysgeusia, and insomnia. Because the topiramate component has teratogenic potential, the combination product carries a black box warning. The doses of both phentermine and topiramate in the extended release combination tablet are lower than those approved for the individual components. Thus, the reported adverse events with phentermine-topiramate CR are manageable, and discontinuation rates are lower than placebo.

## 10. Principle 9—Bariatric Surgery Should Only Be Considered after Failure of a Formal Medical Management Program for Overweight and Obesity

**Figure 2:**
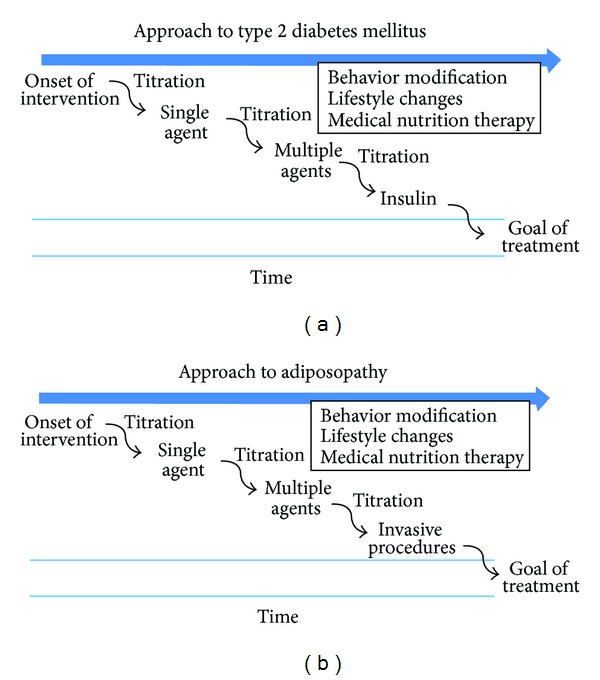
Models of chronic disease management as a practical future approach to treatment of adiposity and adiposopathy [8].

This paper does not deal with the surgical management of overweight and obesity. Consideration of referral for bariatric surgery must always take place only after patients have had the benefit of the team approach to weight management, including behavior modification and pharmacotherapy. In our hands, most patients meet the goal of 5–10% body weight loss when we approach overweight and obesity like we approach any other chronic diseases. Overweight or obesity refractory to medical management does warrant consideration of bariatric surgery (Figure 2) [8].

## 11. Principle 10—A Major Goal of Treatment Should Be to Return Adipose Tissue Function toward Normal in Patients with Overweight and Obesity

The primary goal in the management of overweight and obesity is to correct their complications. A parallel goal is to return adipose tissue function to normal through effective weight loss. Any weight loss will improve the fat-mass (adiposity) related complications. When weight loss is achieved, it is usually a global loss of fat mass. Thus, fat depots like intra-abdominal and visceral fat are also effectively reduced in most cases [8, 12]. In some individuals, however, visceral fat mass is preserved, and the metabolic disorders associated with it persist [69, 130–135]. For this reason, ongoing monitoring is necessary for all patients. Resolution of laboratory abnormalities or signs and symptoms due to adiposopathy (e.g., resumption of regular menstrual flows in women with polycystic ovarian syndrome) should be the desired outcome, not just the loss of pounds of body weight.

## 12. Conclusion

This paper defines the principles of medical practice in bariatric endocrinology—the treatment of overweight and obesity as a means to treat adiposopathy and its accompanying metabolic and hormonal derangements. Overweight and obesity are a continuum and together represent a chronic, biological preventable and treatable disease. The goal of treatment is not only to decrease fat mass, but also to return adipose tissue dysfunction to normal. The principles of chronic disease management that apply to other chronic diseases also apply to the management of overweight, obesity, and adiposopathy.

## Figures and Tables

**Table 1:** Some complications of obesity by body system.

Cardiovascular	
(i) Hypertension	
(ii) Congestive heart failure	
(iii) Cor pulmonale	
(iv) Varicose veins	
(v) Peripheral edema	
(vi) Pulmonary embolism	
(vii) Coronary artery disease	
Endocrine (adiposopathy)	
(i) The metabolic syndrome	
(ii) Type 2 diabetes/hyperinsulinemia	
(iii) Dyslipidemia	
(iv) Polycystic ovarian syndrome/androgenicity	
(v) Amenorrhea/infertility/menstrual disorders	
(vi) Hyperleptinemia/hypoadiponectinemia	
Gastrointestinal	
(i) Gastroesophageal reflux disease (GERD)	
(ii) Nonalcoholic fatty liver disease (NAFLD)	
(iii) Cholelithiasis	
(iv) Hernias	
(v) Colon cancer	
Genitourinary	
(i) Urinary stress incontinence	
(ii) Obesity-related glomerulopathy	
(iii) Hypogonadism (male)	
(iv) Breast and uterine cancer	
(v) Pregnancy complications	
Integument	
(i) Striae distensae (stretch marks)	
(ii) Stasis pigmentation of legs	
(iii) Lymphedema	
(iv) Cellulitis	
(v) Intertrigo, carbuncles	
(vi) Acanthosis nigricans/skin tags	
Musculoskeletal	
(i) Hyperuricemia and gout	
(ii) Immobility	
(iii) Osteoarthritis (knees, hips)	
(iv) Low back pain	
Neurologic	
(i) Stroke	
(ii) Idiopathic intracranial hypertension	
(iii) Meralgia paresthetica	
Psychological	
(i) Depression/low self esteem	
(ii) Body image disturbance	
(iii) Social stigmatization	
Respiratory	
(i) Dyspnea	
(ii) Obstructive sleep apnea	
(iii) Hypoventilation syndrome	
(iv) Pickwickian syndrome	
(v) Asthma	

**Table 2:** Second-generation antipsychotics and the risk of metabolic abnormalities [47, 49].

Drug	Weight gain	Risk for diabetes mellitus	Worsening lipid profile
Clozapine	+++	+	+
Olanzapine	+++	+	+
Risperidone	++	D	D
Quetiapine	++	D	D
Aripiprazole*	+/−	−	−
Ziprasidone*	+/−	−	−

+: increase effect; −: no effect; D: discrepant results; *newer drugs with limited data.

**Table 3:** Selected medications associated with increased adipose tissue mass.

Psychiatric/neurological	
(i) Antipsychotic agents: phenothiazine, olanzapine, clozapine, risperidone	
(ii) Mood stabilizers: lithium	
(iii) Antidepressants: tricyclics, monoamine oxidase inhibitors, selective serotonin reuptake inhibitors (paroxetine hydrochloride), mirtazapine	
(iv) Antiepileptic drugs: gabapentin, valproate sodium, carbamazepine	
Steroid hormones	
(i) Corticosteroids	
(ii) Progestational steroids	
Antidiabetes agents	
Insulin, sulfonylureas, thiazolidinediones	
Antihypertensive agents	
Beta- and alpha-1 adrenergic receptor blockers	
Antihistamines	
Cyproheptadine hydrochloride	
HIV protease inhibitors	
Lipodystrophy (central obesity)	

**Table 4:** Laboratory findings in patients with adiposopathy.

(i) Elevated leptin	
(ii) Decreased adiponectin	
(iii) Increasing leptin to adiponectin ratio over time	
(iv) Increased tumor necrosis factor alpha	
(v) Increased c-reactive protein	
(vi) High triglycerides/low HDL cholesterol	
(vii) Elevated free fatty acids	
(viii) Hyperinsulinemia/hyperglycemia	
(ix) Activation of renin-angiotensin-aldosterone	
(x) Hypoandrogenemia in men	
(xi) Hyperandrogenemia in women	

**Table 5:** Incidence of metabolic disorders by BMI category.

Comorbidity	Overall population with comorbidity, %	Subjects in BMI ranges (kg/m^2^), %					
		18.5–24.9	25.0–26.9	27.0–29.9	30.0–34.9	35.9–39.9	≥40
Diabetes*	8.9	4.2	5.7	10.1	12.1	16.4	27.2
Hypertension^†^	28.9	17.6	25.3	30.8	39.3	44	51.3
Dyslipidemia^‡^	52.9	38.2	53.1	62.2	68	67.5	62.5

*Includes previously diagnosed and undiagnosed diabetes mellitus. Diagnosed is based on self-report. Undiagnosed is defined using the criterion of fasting glucose >125 mg/dL.

^†^Elevated blood pressure (systolic ≥140 mm Hg, or diastolic ≥90 mm Hg), or taking antihypertensive medications.

^‡^Any of the following: total cholesterol ≥240 mg/dL, triglycerides >200 mg/dL, low-density lipoprotein ≥160 mg/dL, or high-density lipoprotein <40 mg/dL.

Adapted from: [62].

**Table 6:** Management model for diabetes and obesity: a successful model of care.

Diabetes mellitus	Overweight and obesity
Initial workup/risk assessment	Initial workup/risk assessment
Institute treatment	Institute treatment
Patient and family education	Patient and family education
Glucose self-monitoring	Step count and weekly weight
Quarterly office assessments: follow A1C, BMI, BP	Quarterly office assessments: follow BMI, BP, WC
Periodic screening for complications and risk restratification	Periodic screening for complications and risk restratification

A1C: glycosylated hemoglobin A1C; BMI: body mass index; BP: blood pressure; WC: waist circumference.

See [63].
